# Optimal Vitamin D Supplementation Doses that Minimize the Risk for Both Low and High Serum 25-Hydroxyvitamin D Concentrations in the General Population

**DOI:** 10.3390/nu7125527

**Published:** 2015-12-04

**Authors:** Paul J. Veugelers, Truong-Minh Pham, John Paul Ekwaru

**Affiliations:** School of Public Health, University of Alberta, 350 University Terrace, Edmonton, AB T6G 2T4, Canada; ptrminh@gmail.com (T.-M.P.); ekwaru@ualberta.ca (J.P.E.)

**Keywords:** vitamin D, vitamin D deficiency, recommended daily allowance, vitamin D supplementation, disease prevention, public health, nutrition, optimal vitamin D intake, optimal vitamin D status

## Abstract

The Recommended Dietary Allowance (RDA) is the nutrient intake considered to be sufficient to meet the requirements of 97.5% of the population. Recent reports revealed a statistical error in the calculation of the RDA for vitamin D opening the question of what the recommendation should be. We took a dual approach to answer this question: (1) we aggregated 108 published estimates on vitamin D supplementation and vitamin D status; and (2) we analyzed 13,987 observations of program participants. The aggregation of published data revealed that 2909 IU of vitamin D per day is needed to achieve serum 25-hydroxyvitamin D (25(OH)D) concentrations of 50 nmol/L or more in 97.5% of healthy individuals. For normal weight, overweight and obese program participants this was 3094, 4450 and 7248 IU respectively. These supplementation doses would also result in 2.5% of normal weight, overweight and obese participants having 25(OH)D concentrations above 210, 200 and 214 nmol/L respectively. As these concentrations are high, an approach that minimizes the risk for both low and high concentrations seems desirable. With this approach we estimated, for example, that doses of 1885, 2802 and 6235 IU per day are required for normal weight, overweight and obese individuals respectively to achieve natural 25(OH)D concentrations (defined as 58 to 171 nmol/L). In conclusion, the large extent of variability in 25(OH)D concentrations makes a RDA for vitamin D neither desirable nor feasible. We therefore propose recommendations be articulated in the form of an optimal intake that minimizes the risk for both low and high serum 25(OH)D concentrations. This contribution includes body weight specific recommendations for optimal intakes for various combinations of lower and upper 25(OH)D concentration targets.

## 1. Introduction

To ensure adequate intake the Institute of Medicine (IOM) issues nutritional recommendations. The Recommended Dietary Allowance (RDA) is the nutrient intake considered to be sufficient to meet the requirements of 97.5% of healthy individuals [1]. The current RDA for vitamin D is 600 IU per day for individuals 1 to 70 years of age and 800 IU per day for those above the age of 70 years [1]. This RDA is assumed to achieve adequate serum 25-hydroxyvitamin D (25(OH)D) concentrations in 97.5% of healthy individuals. Serum 25-hydroxyvitamin D (25(OH)D) is the established marker for vitamin D status.

The RDA for vitamin D has caused some controversy in the past [2,3]. One concern relates to the fact that the RDA is based on aggregations of studies that included clinical subgroups with compromised vitamin D status whereas the RDA is issued for the general population. Another concern relates to the fact that the RDA is uniform across body weight groups, whereas several studies have suggested that recommendations for supplementation should be body weight specific whereby obese individuals may need 2 to 3 times higher doses than normal weight subjects [4,5,6,7]. Further, some recent studies revealed the concern that despite compliance with recommended supplementation doses, serum 25(OH)D targets were still not met and vitamin D deficiency remained prevalent [8,9]. Most recently, reports from Canada and the US reported and confirmed a statistical error in the estimation of the RDA for vitamin D [10,11]. As it appears, the variability in serum 25(OH)D concentrations in response to vitamin D intake is much larger than was assumed [10,11]. This gives rise to the concern that recommendations may leave some subjects vitamin D deficient whereas others following the same recommendations may in fact respond with undesirable high serum 25(OH)D concentrations.

The objective of the present study is to characterize the optimal vitamin D supplementation needed to achieve 25(OH)D targets for the general population. In other words, how much vitamin D should we supplement to maximize adequate serum 25(OH)D concentrations while ensuring these concentrations are not getting too high. This study is not about advising on supplementation to achieve specific health benefits nor about revealing biological mechanisms.

## 2. Methods

We used two approaches to characterize the vitamin D supplementation needed to achieve 25(OH)D targets for the general population: (1) We searched the literature for studies reporting dose response relationships between vitamin D supplementation and serum 25(OH)D concentrations and extracted the reported means and standard deviations (SD) of the serum 25(OH)D response. We included studies for which the mean age of participants was between 1 and 70 years, and we excluded studies among pregnant women. Based on the extracted means and SD’s, we estimated the dose response relationship and lower and upper limits of the prediction interval: the lower limit, the 2.5th percentiles, as mean − 1.96 × SD, and the upper limit, the 97.5th percentiles, as mean + 1.96 × SD. For this purpose we fitted a mixed effects regression model for the mean, the 2.5th and the 97.5th percentiles by vitamin D supplementation doses, weighting by the inverse of the squared standard errors of the respective mean responses. We applied a model with both an exponential and a linear term for the dose response relationship as this model provided the best fit [12]. We stratified these analyses to those with and without potentially compromised vitamin D status (e.g., were selected vitamin D deficiency cases, institutionalized, or hospitalized for long periods). We further stratified by latitude, type of vitamin D (D2 or ergocalciferol *versus* D3 or cholecalciferol), and duration of supplementation to illustrate their importance for the dose response relationship between vitamin D intake and serum 25(OH)D concentrations; (2) For our second approach we accessed and analyzed recordings of 25(OH)D concentrations from 18 to 70 years old healthy volunteer participants of a preventive health program provided by the Canada based Pure North S’Energy Foundation (PN), a not-for-profit charitable organization providing free services since October 2007. The program employs health professionals who provide informed lifestyle counseling [13,14]. At enrollment, participants complete a lifestyle questionnaire, have a medical history and biometric measurements taken (height, weight, waist circumference, blood pressure) and have blood drawn for the assessment of serum 25(OH)D. The collected information serves the purpose of informing the health professionals as a basis for the lifestyle counseling. This counseling includes customized recommendations on diet, physical activity, sleep and stress management. Dietary supplementation is often encouraged and vitamin D supplementation in particular given Canada’s Northern latitude, limited sunlight and limited cutaneous synthesis of vitamin D. Follow up visits for health assessments and lifestyle counseling are scheduled annually. At baseline and each follow up visit health professionals interview the participants and record the amount of vitamin D supplementation participants have been taken. The primary objective of the PN program is lifestyle counseling and disease prevention rather than scientific research. However, the PN does make their collected data available, in anonymized form, to the University of Alberta to allow for secondary data analysis. For that purpose, participants signed and granted written informed consent to allow their relevant information to be used for secondary data analysis. More than 93% of participants in this program resided in latitudes above 50° N (median = 51.48° N). Since inception of the PN program and till June 2013, serum 25(OH)D concentrations were assessed with an automated chemiluminescent immunoassay from DiaSorin (LIAISON) at the Calgary Laboratory Services (coefficient of variation (CV) of inter-assay was 11%), and after this date with liquid chromatography-tandem mass spectrometry (LC-MS/MS) at Doctor’s Data (CV of inter-assay was 2.4% and CV of intra-assay was 1.7%). Both are accredited laboratories and both participate in proficiency surveys (DEQAS).

We excluded baseline records for which participants had indicated that they had taken vitamin D supplements rarely or for less than 3 months. This left us with a dataset of 11,693 participants with a total of 13,987 25(OH)D assessments and recordings of vitamin D supplementation. The supplementation ranged from 0 to 32,000 IU per day. Though all supplementation values were considered in the statistical analyses, the figures depict only levels up to 20,000 IU because only 48 observations were in excess of 20,000 IU. We used quantile regression to model the effect of vitamin D supplementation on the 2.5th percentile, the median and the 97.5% percentile of serum 25(OH)D concentrations. While traditional regression methods model the effects of the covariates on the mean of the dependent variable, quantile regression, which was introduced by Koenker and Bassett [15], extends this and allows modeling of the effects of covariates on different sections (percentiles) of the distribution of the dependent variable. We applied an exponential model as this model provided the best fit for these data and obtained confidence intervals (CI) for the estimates through bootstrapping with 500 replications. In addition, we used logistic regression to estimate the probability of having serum levels above a lower and below an upper serum 25(OH)D concentration. In this respect, we provide an example of having natural serum concentrations, defined as 58 to 171 nmol 25(OH)D/L [16]. We further provide these estimates for normal weight, overweight and obese participants. In these logistic regression models a log term of supplementation provided the best fit. To include zero supplementation levels, the log transformation requires an addition of a constant to supplementation levels. Instead of adding an arbitrary constant, we include this constant as an additional term in the model that was also estimated by the model fitting procedure.

PN anonymized their data prior to forwarding it to the University of Alberta for analyses. The Human Research Ethics Board of the University of Alberta approved access to and analysis of the PN data for the purpose of the present analyses. All analyses were conducted using SAS 9.4 (SAS Institute, Cary, NC, USA). Statistical significance was defined as a *p*-value of less than 0.05.

## 3. Results

**Figure 1:**
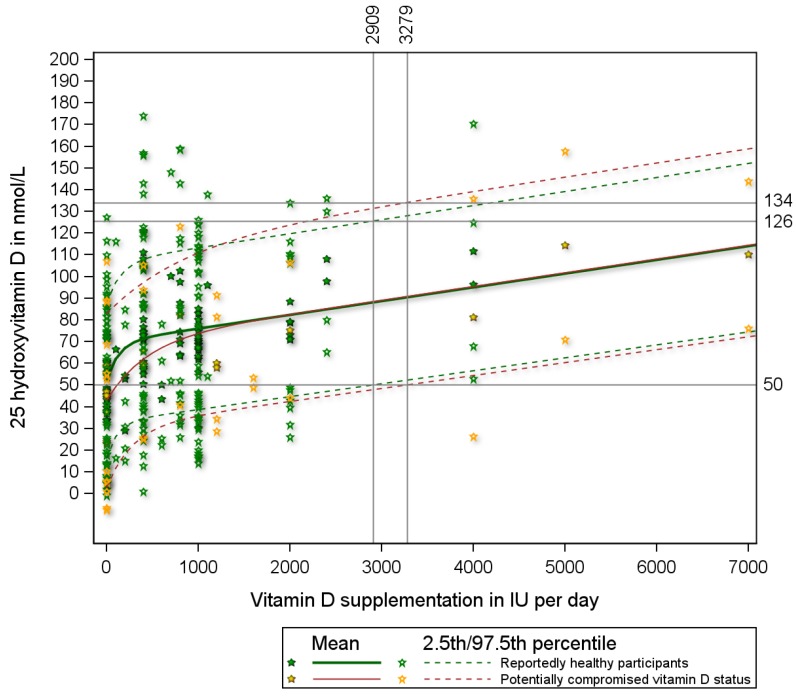
The mean and 95% prediction interval (2.5th and 97.5th percentiles) of serum 25(OH)D concentrations by vitamin D supplementation derived from published study means and standard deviations. Note: The 2.5 percentile line depicts 25(OH)D concentrations for any given vitamin D supplementation dose with 2.5% of all expected 25(OH)D responses to have values below the line, and 97.5% to have values above the line. The 97.5 percentile line depicts 25(OH)D concentrations for any given vitamin D supplementation dose with 97.5% of all expected 25(OH)D responses to have values below the line, and 2.5% to have values above the line. The area between the 2.5 percentile line and the 97.5 percentile line depicts the 95% prediction interval of 25(OH)D concentrations for any given vitamin D supplementation dose. The equation for the mean response for reportedly healthy individuals is *Y* = 51.9 + 17.7 *(1 − e^−7.4*X^) + 6.3 *X in which Y denotes serum 25(OH)D (in nmol/L) and *X* vitamin D supplementation (in 1000 IUs).

We retrieved a total of 36 published studies [17,18,19,20,21,22,23,24,25,26,27,28,29,30,31,32,33,34,35,36,37,38,39,40,41,42,43,44,45,46,47,48,49,50,51,52] that reported both the mean and standard deviations of serum 25(OH)D responses for a total of 108 study doses. The appendix lists these 36 studies and provides details on participants’ characteristics, study size, supplementation dose, and mean and standard deviation of the serum 25(OH)D concentrations. The mean serum 25(OH)D concentrations reported in these studies ranged from 23 to 240 nmol/L. The standard deviations ranged from 6.6 to 97.5, had a mean value of 19.9 and a median value of 17.8 nmol/L. Of the 108 study doses, 91 were reportedly given to healthy participants and 17 to participants with potentially compromised vitamin D status. When calculating the 2.5th percentiles, three of the 108 study doses revealed negative values for serum 25(OH)D and were therefore excluded. Figure 1 presents the dose response relationship between vitamin D supplementation and serum 25(OH)D with the solid lines representing the fitted relationship for the mean and the dashed lines representing the fitted relationships for the 2.5th and 97.5th percentiles (the estimated 95% prediction interval). The regression line started off lower for studies among participants with potentially compromised vitamin D status relative to studies among reportedly healthy participants, but for supplementation doses above 1000 IU per day, the dose response relationship is similar for both groups (Figure 1). To achieve serum 25(OH)D concentrations of 50 nmol/L or more in 97.5% of reportedly healthy individuals, a dose of at least 2909 (95% CI: 1962–10,847) IU per day is needed (Figure 1). At this supplementation dose (2909 IU per day) 2.5% of healthy individuals reach serum 25(OH)D concentrations in excess of 126 nmol/L (Figure 1). Those with compromised vitamin D status need at least 3279 (1668–8740) IU per day to achieve serum 25(OH)D concentrations of 50 nmol/L or more in 97.5% of this subgroup. To achieve serum 25(OH)D concentrations of 40 nmol/L or more in 97.5% of reportedly healthy individuals, supplementation with at least 1229 (569–2819) IU per day is needed (Figure 1).

The dose response relationship for reportedly healthy participants is further characterized by latitude, by type of vitamin D supplementation (vitamin D2 *versus* D3), and by the period between commencing supplementation and assessing serum 25(OH)D concentrations (supplementary figures S1, S2 and S3 respectively). Relative to studies executed in regions between 50° S and 50° N, participants of studies above 50° N had 25(OH)D concentrations that were on average approximately 10 nmol/L lower, and participants of studies below 50° S had concentrations that were approximately 20 nmol/L lower (Supplementary Figure S1). Supplementary Figure S2 depicts the dose response relationships for studies examining vitamin D3 supplementation exclusively and for the compilation of studies examining either vitamin D2, vitamin D3 or unspecified vitamin D types. Too few studies had examined vitamin D2 to allow for a direct comparison of vitamin D2 with vitamin D3. The fact that these dose response relationships are almost identical suggests the dose response relationships for vitamin D2 and vitamin D3 may be similar. Supplementary Figure S3 depicts the dose response relationships by period between commencing supplementation and assessing serum 25(OH)D concentrations. Also here, too few studies with a short period between commencing supplementation and assessment of serum 25(OH)D concentrations were available to allow for stratification by the length of this period. The difference between the dose response relationships depicted in supplementary Figure S3 suggests that too short a period between commencing supplementation and assessing serum 25(OH)D concentrations lead to an underestimation of serum 25(OH)D for any given supplementation dose.

**Figure 2:**
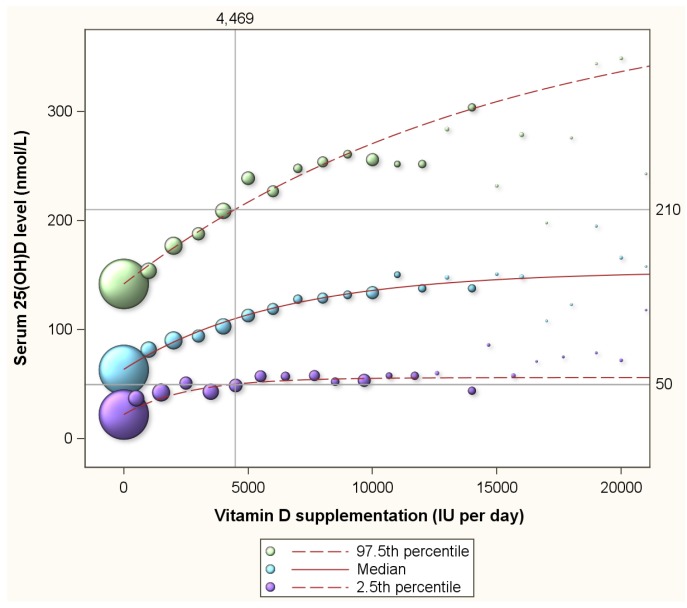
The median and 2.5th and 97.5th percentiles of serum 25(OH)D concentrations by vitamin D supplementation among 11,693 healthy participants of a preventive health program in Canada. Note: The 2.5 percentile line depicts 25(OH)D concentrations for any given vitamin D supplementation dose with 2.5% of all expected 25(OH)D responses to have values below the line, and 97.5% to have values above the line. The 97.5 percentile line depicts 25(OH)D concentrations for any given vitamin D supplementation dose with 97.5% of all expected 25(OH)D responses to have values below the line, and 2.5% to have values above the line. The area between the 2.5 percentile line and the 97.5 percentile line depicts the 95% prediction interval of 25(OH)D concentrations for any given vitamin D supplementation dose. The median, the 2.5th and 97.5th percentiles were estimated through quantile regression, a statistical approach that accommodates the clearly visible skewness in the distribution of 25(OH)D concentrations.

Table 1 presents a summary of the 13,987 assessments of the 11,693 program participants. Of all the assessments, 33.2%, 1.4%, 36.7% and 28.8% were from normal weight, underweight, overweight and obese participants respectively (Table 1). Figure 2 depicts the 2.5th percentile, the median and 97.5th percentile of serum 25(OH)D concentrations in response to supplementation ranging from 0 to 20,000 IU per day. The 2.5th percentile of 50 nmol/L is achieved with supplementation intake of 4469 (3607–6015) IU per day (Figure 2). At this supplementation levels, 2.5% of participants reach serum 25(OH)D concentrations in excess of 210 nmol/L. For normal weight, overweight and obese subgroups, supplementation with 3094 (2288–3658) IU, 4450 (2977–13,976) IU and 7248 (4770–10,797) IU, respectively, are estimated to be required to achieve a 2.5th percentile of 50 nmol/L respectively. At these supplementation intakes, 2.5% of the individuals would reach serum 25(OH)D levels in excess of 210 nmol/L, 200 nmol/L, and 214 nmol/L, repectively. Figure 2 further reveals that the 2.5th percentile line does not exceed 60 nmol/L for the depicted supplementation range of 0 to 20,000 IU per day.

**Table 1:** Summary of 13,987 simultaneous assessments of reported vitamin D supplementation and serum 25(OH)D level from 11,693 participants.

	*N*	*%*	*Mean*	*Std*
Vitamin D supplementation (IU per day)	13,987		2484.6	3993.8
Plasma 25(OH)D level nmol/L	13,987		88.9	47.6
**Age (Years)**				
<40	5270	37.7		
40 to 49	3123	22.3		
50 to 59	3528	25.2		
60+	2066	14.8		
**Gender**				
Female	6760	48.3		
Male	7227	51.7		
**Weight Status**				
Normal weight	4470	33.1		
Underweight	184	1.4		
Overweight	4943	36.7		
Obesity	3888	28.8		
**Season**				
Winter	4685	33.5		
Spring	4198	30.0		
Summer	2717	19.4		
Fall	2387	17.1		

**Figure 3:**
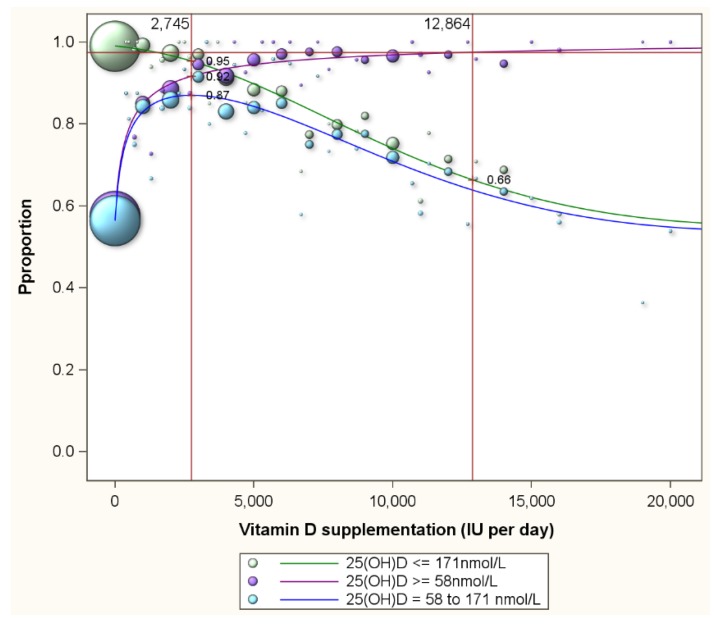
Proportion of participants with serum 25(OH)D concentrations above the lower natural limit, below the upper natural limit and within the natural range. Note: The purple line represents the proportion of individuals with serum 25(OH)D concentrations of 58 nmol/L or more by vitamin D supplementation level; the green line the proportion of participants with concentrations of 171 nmol/L or less; and the blue line the proportion of participants that have serum concentrations above 58 nmol/L and below 171 nmol/L. The blue line is curved and shows that the proportion above 58 nmol/L and below 171 nmol/L peaks at 87% at a supplementation dose of 2745 IU per day. At this optimal dose, 8% (100%–92%) will have serum 25(OH)D concentrations below 58 nmol/L and 5% (100%–95%) in excess of 171 nmol/L.

Luxwolda *et al.* [16] had revealed that natural 25(OH)D concentrations range from 58 to 171 nmol/L. Figure 3 presents the proportion of individuals with serum 25(OH)D concentrations of 58 nmol/L or more by vitamin D supplementation level (the purple line) and the proportion of participants with concentrations of 171 nmol/L or less (the green line). Clearly, with increasing supplementation doses, the proportion of participants with serum 25(OH)D concentrations above 58 nmol/L increases and the proportion with concentrations below 171 nmol/L decreases. Supplementation of 12864 (9930–19,067) IU per day is needed to achieve that 97.5% of participants reach 58 nmol/L or more. At this supplementation level 34% of participants will reach serum 25(OH)D concentrations in excess of the upper limit of the natural range (171 nmol/L). The blue line in Figure 3 represents the proportion of participants that have serum concentrations above 58 nmol/L and below 171 nmol/L. This line is curved and shows that the proportion above 58 nmol/L and below 171 nmol/L peaks at 87% at a supplementation dose of 2745 (2616–3147) IU per day. This peak represents the optimal supplementation dose. At this optimal dose, 8% will have serum 25(OH)D concentrations below 58 nmol/L and 5% in excess of 171 nmol/L (Figure 3).

**Figure 4:**
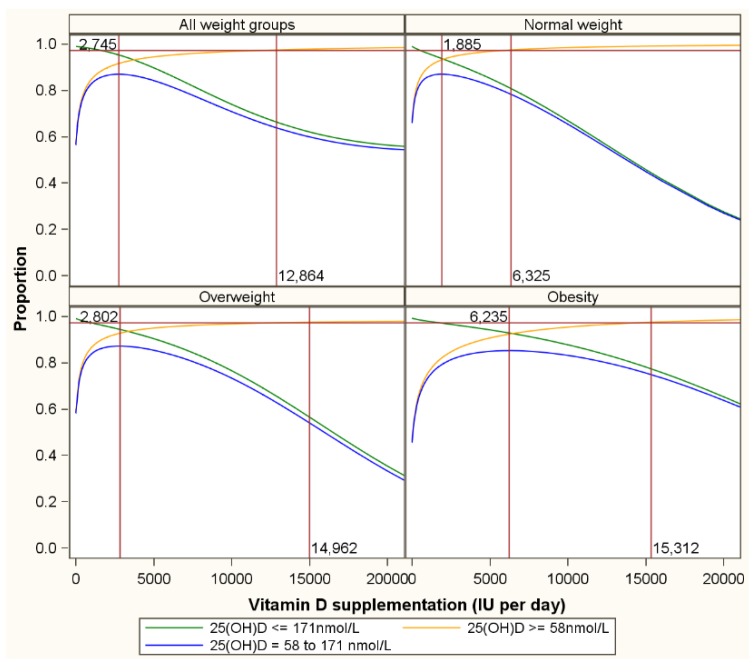
Proportion of participants with serum 25(OH)D concentrations above the lower natural limit, below the upper natural limit and within the natural range by body weight status. Note: The orange lines represent the proportion of individuals with serum 25(OH)D concentrations of 58 nmol/L or more by vitamin D supplementation level; the green lines the proportion of participants with concentrations of 171 nmol/L or less; and the blue lines the proportion of participants that have serum concentrations above 58 nmol/L and below 171 nmol/L.

Figure 4 is a repeat of Figure 3 for normal weight, overweight and obese subgroups. Optimum proportions of participants with natural serum 25(OH)D concentrations are reached with supplementation with 1885 (1518–2166), 2802 (2400–3666) and 6235 (5943–7004) for normal weight, overweight and obese participants respectively. The lower target of 58 nmol/L and the upper target of 171 nmol/L applied in figures 3 and 4 were just examples to illustrate the concept. Table 2 presents for several combinations of lower and upper serum 25(OH)D targets the vitamin D supplementation needed to maximize the probability to achieve serum 25(OH)D concentrations between the lower and upper target.

**Table 2:** Vitamin D supplementation that maximizes the proportion of participants with serum 25(OH)D concentrations within various lower and upper serum 25(OH)D targets.

			*LOWER SERUM 25(OH)D TARGETS (in nmol/L)*																							
			40				50				58				75				100				150			
				*Proportion*				*Proportion*				*Proportion*				*Proportion*				*Proportion*				*Proportion*		
		*Weight Status*	*Optimal Intake (IU)*	*Between Lower and Upper*	*Above Lower*	*Below Upper*	*Optimal Intake (IU)*	*Between Lower and Upper*	*Above Lower*	*Below Upper*	*Optimal Intake (IU)*	*Between Lower and Upper*	*Above Lower*	*Below Upper*	*Optimal Intake (IU)*	*Between Lower and Upper*	*Above Lower*	*Below Upper*	*Optimal Intake (IU)*	*Between Lower and Upper*	*Above Lower*	*Below Upper*	*Optimal Intake (IU)*	*Between Lower and Upper*	*Above Lower*	*Below Upper*
*Upper Serum 25(OH)D Targets (in nmol/L)*	100	Normal weight	**104**	0.70	0.91	0.79	**238**	0.63	0.87	0.76	**377**	0.56	0.83	0.73	**837**	0.36	0.70	0.66								
		Overweight	**129**	0.74	0.90	0.84	**301**	0.65	0.85	0.79	**513**	0.57	0.81	0.75	**1108**	0.36	0.69	0.67								
		Obesity	**364**	0.77	0.91	0.87	**755**	0.66	0.84	0.82	**1177**	0.55	0.77	0.77	**2603**	0.31	0.64	0.67								
		**All weight groups**	**349**	**0.77**	**0.93**	**0.84**	**552**	**0.69**	**0.88**	**0.81**	**719**	**0.61**	**0.82**	**0.79**	**1048**	**0.41**	**0.65**	**0.75**								
	125	Normal weight	**323**	0.85	0.95	0.90	**606**	0.79	0.92	0.87	**886**	0.74	0.89	0.85	**1798**	0.58	0.79	0.79								
		Overweight	**622**	0.88	0.96	0.93	**939**	0.83	0.92	0.91	**1209**	0.78	0.88	0.90	**1808**	0.61	0.75	0.86								
		Obesity	**830**	0.87	0.94	0.93	**1717**	0.79	0.90	0.89	**2683**	0.71	0.85	0.85	**4960**	0.52	0.74	0.78								
		**All weight groups**	**669**	**0.88**	**0.96**	**0.93**	**1025**	**0.82**	**0.92**	**0.91**	**1322**	**0.76**	**0.87**	**0.89**	**1941**	**0.59**	**0.73**	**0.86**	**4138**	**0.27**	**0.57**	**0.70**				
	150	Normal weight	**499**	0.91	0.96	0.95	**889**	0.87	0.94	0.93	**1280**	0.82	0.91	0.91	**2650**	0.68	0.83	0.85	**6208**	0.44	0.74	0.71				
		Overweight	**731**	0.91	0.96	0.95	**1274**	0.86	0.93	0.93	**1779**	0.82	0.90	0.92	**3156**	0.68	0.81	0.87	**6479**	0.42	0.67	0.76				
		Obesity	**1620**	0.92	0.97	0.96	**3181**	0.87	0.94	0.93	**4640**	0.81	0.90	0.91	**7250**	0.66	0.80	0.86	**9862**	0.40	0.61	0.80				
		**All weight groups**	**1012**	**0.93**	**0.97**	**0.96**	**1535**	**0.88**	**0.94**	**0.95**	**1978**	**0.83**	**0.90**	**0.94**	**2949**	**0.68**	**0.78**	**0.90**	**5643**	**0.44**	**0.65**	**0.78**				
	171	Normal weight	**774**	0.94	0.97	0.97	**1340**	0.90	0.95	0.95	**1885**	0.87	0.93	0.94	**3656**	0.75	0.86	0.89	**7209**	0.55	0.78	0.78				
		Overweight	**1276**	0.94	0.97	0.97	**2114**	0.91	0.95	0.96	**2802**	0.87	0.93	0.94	**4611**	0.76	0.85	0.91	**8270**	0.55	0.73	0.82				
		Obesity	**2243**	0.94	0.97	0.97	**4344**	0.90	0.95	0.95	**6235**	0.85	0.92	0.93	**9451**	0.73	0.84	0.89	**12,843**	0.50	0.67	0.83				
		**All weight groups**	**1414**	**0.95**	**0.97**	**0.98**	**2133**	**0.91**	**0.95**	**0.96**	**2745**	**0.87**	**0.92**	**0.95**	**4108**	**0.74**	**0.82**	**0.92**	**6829**	**0.54**	**0.70**	**0.84**	**10,373**	**0.14**	**0.41**	**0.73**
	200	Normal weight	**1199**	0.96	0.98	0.98	**2038**	0.94	0.97	0.97	**2849**	0.91	0.95	0.96	**5479**	0.82	0.90	0.92	**10,008**	0.69	0.86	0.83				
*Upper Serum 25(OH)D Targets (in nmol/L)*		Overweight	**2082**	0.96	0.98	0.98	**3321**	0.94	0.96	0.97	**4250**	0.91	0.94	0.96	**6702**	0.82	0.88	0.94	**11,120**	0.67	0.80	0.87				
		Obesity	**3527**	0.96	0.98	0.98	**6386**	0.94	0.97	0.97	**8714**	0.90	0.95	0.95	**12,365**	0.80	0.88	0.92	**16,112**	0.60	0.73	0.87				
		**All weight groups**	**1883**	**0.96**	**0.98**	**0.98**	**3243**	**0.93**	**0.96**	**0.97**	**4491**	**0.90**	**0.94**	**0.96**	**7297**	**0.80**	**0.87**	**0.93**	**9423**	**0.66**	**0.76**	**0.90**	**12,627**	**0.30**	**0.46**	**0.84**
	250	Normal weight	**2319**	0.98	0.99	0.99	**3971**	0.96	0.98	0.98	**5433**	0.95	0.97	0.98												
		Overweight	**3980**	0.98	0.99	0.99	**5981**	0.96	0.98	0.99	**7281**	0.94	0.96	0.98												
		Obesity	**5433**	0.98	0.99	0.99	**11,402**	0.96	0.99	0.98	**16,917**	0.95	0.98	0.97												
		**All weight groups**	**3543**	**0.98**	**0.99**	**0.99**	**5918**	**0.96**	**0.98**	**0.98**	**7914**	**0.94**	**0.96**	**0.98**	**11,972**	**0.87**	**0.92**	**0.96**	**11,922**	**0.74**	**0.78**	**0.96**	**15,623**	**0.43**	**0.50**	**0.93**
	300	Normal weight	**3307**	0.99	0.99	1.00	**5228**	0.98	0.98	0.99																
		Overweight	**5,042**	0.98	0.99	0.99	**7433**	0.97	0.98	0.99																
		Obesity	**8767**	0.99	0.99	1.00	**13,065**	0.98	0.99	0.99																
		**All weight groups**	**5163**	**0.98**	**0.99**	**0.99**	**8225**	**0.97**	**0.98**	**0.99**	**10,560**	**0.96**	**0.97**	**0.99**	**14,955**	**0.90**	**0.93**	**0.97**	**13,049**	**0.77**	**0.79**	**0.98**	**16,859**	**0.47**	**0.51**	**0.96**

Note: Where the number of observations with serum 25(OH)D concentrations was limited we were not able to provide accurate estimates for weight specific estimates for optimal intake.

## 4. Discussion

The aggregation of published estimates revealed that 2990 IU per day is needed to achieve serum 25(OH)D concentrations of 50 nmol/L or more in 97.5% of healthy individuals. For normal weight participants of the preventive health program this was 3094 IU per day. These estimates are in excess of the current recommendation of 600 IU per day.

Despite the current recommendations, vitamin D deficiency constitutes a tremendous public health burden. According to Statistics Canada the percentage of Canadians having serum 25(OH)D concentrations below 50 nmol/L is 35% [53]. Among Canadians who take vitamin D supplementation, 15% had serum 25(OH)D concentrations below 50 nmol/L [8]. Among older Canadian residing in urban areas and supplementing with 400 IU per day or more, 10% had serum 25(OH)D concentrations below 50 nmol/L [9]. These observations show that the current recommendation is failing the objective of preventing vitamin D deficiency and thus in need of reconsideration. The objective of the present study is to inform this reconsideration. The objective of the present study is not to recommend specific supplementation regimens or specific 25(OH)D targets.

Serum 25(OH)D concentrations are affected by various host and environmental factors including age, sex, diet, sun exposure, physical activity, body weight, skin pigmentation, genetic factors, among others [4,54,55]. Body weight appeared to have a profound effect on the dose response relationship between vitamin intake and 25(OH)D such that overweight and obese individuals are recommended respectively 1.5 and two to three times more vitamin D than normal weight subjects[3,4]. Sun exposure, responsible for the endogenous synthesis of vitamin D, varies by latitude and season. In the present study, we revealed that relative to observations between 50° S and 50° N, participants of studies above 50° N had 25(OH)D concentrations that were on average approximately 10 nmol/L lower. This relative modest variation by latitude seems consistent with observations of others [56,57,58], but may represent an underestimation resulting from the fact that latitude is a poor proxy of actual sun exposure and endogenous synthesis of vitamin D. This may also apply to season as a proxy of sun exposure and endogenous synthesis of vitamin D. Modest variation in 25(OH)D concentrations will also arise from dietary vitamin D as the vitamin D content of diets is relatively low. For example, the Canadian diet of adults contains on average 232 IU of vitamin D [59] and that of children on average 167 IU with fortified foods as the primary contributors [60]. British diets reportedly contain 124 IU and 28 IU per day for meat eaters and vegans respectively [61]. Clearly, such relative small quantities of dietary vitamin D are unlikely to introduce large variability in 25(OH)D concentrations. It has also been suggested that 25(OH)D in meat affects the consumer’s serum 25(OH)D concentration and herewith contributes to the variability in the dose response relationship between vitamin D intake and serum 25(OH)D concentrations [61]. It has further been suggested that the fat content of the diet has a profound effect on the absorption of vitamin D supplementation and herewith may introduce variability in the dose response relationship between vitamin D intake and 25(OH)D concentrations [62,63,64]. Furthermore, in addition to the abovementioned host factors, various genetic factors have been suggested to introduce variability in 25(OH)D concentrations [65,66,67,68,69,70,71,72,73]. We should also recognize that the way studies were executed may affect the study outcomes. The minimum period between commencing supplementation and assessing 25(OH)D concentrations should be in excess of three months as illustrated in the present study. Other aspects of research that may affect outcomes include participants’ compliance with supplementation regimens, reporting bias and the laboratory assessment methods.

Collectively, the abovementioned host and environmental factors are responsible for large variability in serum 25(OH)D concentrations in the general population. Large variability also exists for the bodily responses to vitamin D intake as revealed in the present study. Our literature review had revealed standard deviations of 25(OH)D responses to vitamin D intake for 108 study doses. These standard deviations ranged from 6.6 to 97.5 with an average value of 19.9 and a median value of 17.8 nmol/L (Appendix). This large variation is responsible for the fact that high supplementation doses are needed to ensure that 97.5% of subjects achieve serum 25(OH)D concentrations above relatively modest targets, while other subjects will reach high 25(OH)D concentrations at modest supplementation levels. For example, the present study illustrated that 2.5% of normal weight subjects would reach serum 25(OH)D concentrations above 210 nmol/L with the supplementation dose of 3094 IU per day, which is the dose needed to ensure that 97.5% of subjects have serum levels above 50 nmol/L. Hence, if one judges serum 25(OH)D concentrations in excess of 210 nmol/L as undesirable, a target to achieve serum levels above 50 nmol/L in 97.5% of a population would not be feasible because 2.5% would end up with serum concentrations that some will judge as undesirably high. An approach that seeks an optimal supplementation dose that minimizes both low and high 25(OH)D concentrations seems therefore preferable. The present study is the first to take that approach.

Our literature review had identified standard deviations of 25(OH)D responses to vitamin D intake that ranged from 6.6 to 97.5 nmol/L and had a median value of 17.8 nmol/L [17,18,19,20,21,22,23,24,25,26,27,28,29,30,31,32,33,34,35,36,37,38,39,40,41,42,43,44,45,46,47,48,49,50,51,52]. The standard deviation is also considered in the calculation of the RDA[1]. Specifically, the RDA represents the amount of vitamin D that is two standard deviations above the amount that meets the needs of 50% of the population at large. The IOM, in their calculations of the RDA, had assumed too low a value for this standard deviation by mistakenly using standard error where standard deviation should have been used [1,10,11], and, in their recent announcement, had applied the value of 2 ng/mL (5 nmol/L) as standard deviation which is lower than any existing estimate for standard deviation [74]. The 108 existing estimates range from 6.6 to 97.5 nmol/L with a median value of 17.8 nmol/L [17,18,19,20,21,22,23,24,25,26,27,28,29,30,31,32,33,34,35,36,37,38,39,40,41,42,43,44,45,46,47,48,49,50,51,52]. Clearly, had the IOM used any of the existing standard deviations, they would have estimated a higher RDA. Specifically, the IOM suggested 400 IU per day as the amount of vitamin D that meets the needs of 50% of the population [1]. With the assumption that 400 IU per day achieves an average serum 25(OH)D concentration of 40 nmol/L[1], the present analysis shows that 1229 IU per day is needed to achieve that 97.5% of the population has 25(OH)D concentrations of 40 nmol/L or more. However, others have argued that the recommendation of 400 IU per day as the dose that meets the needs of 50% of the population is too low [2,3,75,76,77]. Higher values for the amount of vitamin D that meet the needs of 50% of the population will come with progressively higher values for the amount that meets the needs for 97.5% of the population.

Benefits of vitamin D supplementation have been suggested for the prevention of fractures in the elderly [78,79], reduction of risk for metabolic syndrome and insulin resistance [13,80], reduction of adverse cardiovascular events [81,82], prevention of cancer [34,83,84,85,86,87,88], multiple sclerosis [89], and improvements in immune function [90,91,92]. Several well powered, long term randomized clinical trials with supplementation doses of up to 3,200 IU per day are currently underway [93]. Where these reveal positive results and where these lead to recommendations to adjust the current RDA, our observations suggest two considerations: (1) that not only a lower serum 25(OH)D target is considered but an upper serum 25(OH)D target as well; and (2) the new recommendations be body weight status specific.

Various studies, including some reported recently, have suggested adverse effects of high serum 25(OH)D concentrations[94,95,96,97,98]. Other recent studies have suggested the absence of associations of vitamin D supplementation and serum 25(OH)D concentrations with hypercalcemia [4,99] adding to interpretations that adverse effects from vitamin D doses of up to 50,000 IU per day are very rare [100,101] and that “vitamin D is not as toxic as was once thought” [102]. In the present study, very few subjects develop 25(OH)D concentrations in excess of 300 nmol/L. For this reason it appeared very challenging to model the probability of targets higher than 300 nmol/L. Whether 300 nmol/L, or lower or higher serum concentrations, should be set as the upper target to ensure safety is beyond the scope of the present study. Likewise, advising on supplementation and 25(OH)D concentrations to achieve specific health benefits, is beyond the scope of the present study. In this respect, a recent review of the literature concluded that specific health conditions may be prevented at distinct serum 25(OH)D concentrations [103].

A strength of the present study is its dual approach that included a meta analysis of randomized controlled trials of vitamin D supplementation and an analysis of a large preventive health program with subjects using a wide range of vitamin D supplementation and with information on various host and environmental factors allowing us to adjust for their confounding influence. We acknowledge that vitamin D studies are difficult to generalize because vitamin D status is determined by so many host and environmental factors. This applies to the randomized controlled trials included in our meta analysis and our analysis of the program participants. As a limitation of the meta analysis we further acknowledge our inability to stratify or adjust for body weight which is an critical determinant. As a limitation of the preventive health program we further acknowledge the presence of selection bias as health conscious individuals are more likely to participate in a volunteer program [14]. Moreover, our meta analysis likely generated biased estimates for the 2.5th and 97.5th percentiles as our analysis of program participants had revealed a high degree of skewness in the distribution of serum 25(OH)D concentrations (Figure 2). This limitation extends to all studies that assume a normal distribution of serum 25(OH)D concentrations. However, despite the abovementioned limitations and sources of bias, estimates for the 2.5th percentiles from our meta analysis and our analysis of the program participants produce relatively similar findings.

## 5. Conclusions

In conclusion, we revealed a very large variation in 25(OH)D concentrations in response to vitamin D intake. This large variation is responsible for the fact that relative high supplementation doses are needed to ensure that the majority of the general population achieves at least modest serum 25(OH)D concentrations while other subjects will reach high 25(OH)D concentrations at modest supplementation levels. For example, where one judges serum 25(OH)D concentrations in excess of 210 nmol/L as undesirably high, a dose recommendation to ensure 25(OH)D concentrations above 50 nmol/L in 97.5% of the general population is not feasible because more than 2.5% of subjects will end up with serum concentrations that some judge as undesirable. We therefore recommend an approach that seeks an optimal supplementation dose that minimizes the risk for both low and high 25(OH)D concentrations. We further recommend these optimal doses be body weight specific.

## Supplementary Materials

Supplementary materials can be accessed at: http://www.mdpi.com/2072-6643/7/12/5527/s1.

## Appendix

Studies that reported both the mean and standard deviation of serum 25(OH)D concentrations by vitamin D intake.

**Table:**

*Study*	*Gender of Participants*	*Vitamin D Supplement*	*Sub Group*	*Dose (IU)*	*Follow Up in Weeks*	*Number of Participants*	*Mean 25(OH)D in nmol/L*	*SD in nmol/L*
Ala-Houhala, M., *et al.*, 1988 [17]	Both	None		0	52	27	43.3	19.5
		Not specified		400	52	24	71.3	23.8
								
Aloia, J., *et al.*, 2010 [18]	Both	D3		4000	13	35	111.7	30.0
								
Aloia, J.F., *et al.*, 2005 [19]	Female	D3		800	13	104	70.8	22.9
Biancuzzo, R.M., *et al.*, 2010 [20]	Both	None		0	11	17	45.3	16.0
		D3	In orange juice	1000	11	16	76.8	21.3
			In capsules	1000	11	18	70.0	27.5
		D2	In orange juice	1000	11	20	66.0	18.5
			In capsules	1000	11	15	68.5	26.3
Bolton-Smith, C., *et al.*, 2007 [21]	Female	None		0	52	55	47.8	17.3
					104	55	48.8	13.3
		D3	Vit D + Calcium	400	52	55	74.3	15.3
					104	55	74.5	15.0
			Vit D + Vit K + Calcium	400	52	58	73.0	16.3
					104	58	70.8	16.5
Cashman, K.D., *et al.*, 2012 [22]	Both	None		0	5	13	39.7	11.1
					10	13	41.2	11.1
		D3		800	5	16	64.1	9.5
					10	16	69.0	8.7
Dawson-Hughes, B., *et al.*, 1991 [23]	Female	None		0	48	121	60.6	28.5
		D3		400	48	125	92.1	23.6
Dawson-Hughes, B., *et al.*, 1995 [24]	Female	Not specified		100	39	124	66.3	25.5
				700	39	123	100.1	24.5
Diamond, T., *et al.*, 2013 [25]	Both	D3		2000	12	11	75.3	15.9
				5000	12	15	114.4	22.2
Gallagher, J.C., *et al.*, 2014 [26]	Both	D3	African American women	2400	52	20	97.8	16.5
			White women	2400	52	5	108.0	14.3
Harris, S.S., *et al.*, 2012 [27]	Both	None		0	12	43	37.4	16.1
		D3		4000	12	46	81.1	27.9
Holick, M.F., *et al.*, 2008 [28]	Both	None		0	11	16	47.0	19.8
		D3		1000	11	20	72.3	27.5
		D2		1000	11	18	67.0	24.0
		D3+D2		1000	11	14	71.0	19.3
Jensen, C., *et al.*, 2002 [29]	Female	Not specified		400	52	22	50.3	19.1
					104	22	80.4	21.6
					156	22	76.6	22.1
Karkkainen, M.K., *et al.*, 2010 [30]	Female	D3		0	156	287	55.9	21.8
				800	156	306	74.6	21.9
Kilpinen-Loisa, P., *et al.*, 2009 [31]	Both	D3		800	26	72	82.0	21.0
Kyriakidou-Himonas, M., *et al.*, 1999 [32]	Female	D3		800	12	10	63.3	11.1
Lagunova, Z., *et al.*, 2013 [33]	Both	D3		2000	4	11	78.9	15.3
Lappe, J.M., *et al.*, 2007 [34]	Female	None		0	52	446	71.1	19.8
		D3		1100	52	288	96.0	21.4
Li-Ng, M., *et al.*, 2009 [35]	Male	D3		2000	12	78	88.5	23.2
Logan, V.F., *et al.*, 2013 [36]	Both	None		0	4	13	71.0	15.3
					8	13	61.0	15.3
					13	13	44.0	15.3
					25	13	37.0	17.9
		D3		1000	4	23	83.0	12.2
					8	23	83.0	14.7
					13	23	80.0	17.1
					25	23	80.0	19.6
		D2		1000	4	25	70.0	12.9
					8	25	66.0	12.9
					13	25	62.0	14.7
					25	25	56.0	11.0
Molgaard, C., *et al.*, 2010 [37]	Female	None		0	26	73	47.0	20.0
					52	73	39.7	17.7
		D3		200	26	74	54.5	12.0
					52	74	52.9	16.3
				400	26	73	59.4	13.2
					52	74	57.9	14.3
Nelson, M.L., *et al.*, 2009 [38]	Female	None		0	52	31	72.7	27.8
		D3		800	52	31	97.4	31.3
Oosterwerff, M.M., *et al.*, 2014 [39]	Both	None		0	8	53	24.0	16.0
					16	53	23.0	15.0
		D3		1200	8	57	58.0	12.0
					16	57	60.0	16.0
								
Orwoll, E.S., *et al.*, 1988 [41]	Male	None		0	26	46	55.0	15.0
					52	46	60.0	18.0
		D3		1000	26	46	75.0	18.0
					52	46	85.0	20.0
Orwoll, E.S., *et al.*, 1989 [40]	Both	None		0	104	14	45.0	22.5
		D3		1600	52	14	240.0	97.5
					104	17	225.0	87.5
Pignotti, G.A., *et al.*, 2010 [42]	Female	None		0	13	29	58.8	24.7
		D3		400	13	29	59.5	17.5
Putman, M.S., *et al.*, 2013 [43]	Both	D3		200	11	25	28.9	7.0
				1000	11	29	30.1	6.6
Rajakumar, K., *et al.*, 2008 [44]	Both	D3	Non-obese	400	4	18	72.8	16.8
			Obese	400	4	21	65.5	20.3
Schaafsma, A., *et al.*, 2000 [45]	Female	D3		400	13	46	108.0	25.0
					26	46	111.0	23.0
					52	46	121.0	27.0
Schnatz, P.F., *et al.*, 2014 [46]	Female	None		0	104	291	45.5	23.7
		D3		400	104	285	60.8	30.5
Schou, A.J., *et al.*, 2003 [47]	Both	None	Placebo then supplementation	0	4	10	33.7	10.4
			Supplementation then placebo	0	4	10	32.3	13.0
		D3	Placebo then supplementation	600	4	10	50.2	14.2
			Supplementation then placebo	600	4	10	43.4	9.2
Smith, S.M., *et al.*, 2009 [48]	Both	None		0	18	18	38.0	17.0
					25	18	34.0	12.0
		D3		400	18	19	55.0	19.0
					25	19	57.0	15.0
				1000	18	18	63.0	20.0
					25	18	63.0	25.0
				2000	18	7	71.0	20.0
					25	7	71.0	23.0
Van Der Klis, F.R., *et al.*, 1996 [49]	Female	None		0	5	41	58.5	10.7
		D3		400	5	19	87.9	28.1
				800	5	19	87.9	28.1
						19	102.6	28.8
Vieth, R., *et al.*, 2001 [50]	Both	D3		1000	12	33	68.7	16.9
				4000	12	28	96.4	14.6
Wamberg, L., *et al.*, 2013 [51]	Both	None		0	26	26	46.8	21.2
		D3		7000	26	26	110.0	17.3
Zwart, S.R., *et al.*, 2011 [52]	Both	Not specified		2000	13	15	73.0	17.0
					26	15	79.0	16.0

Footnote: SD: Standard Deviation: Reported SD ranged from 6.6 to 97.5, had a mean value of 19.9 and a median value of 17.8 nmol/L.
