# Supplementary material for: Optimal Vitamin D Supplementation Doses that Minimize the Risk for Both Low and High Serum 25-Hydroxyvitamin D Concentrations in the General Population
Source: Nutrients. 2015 Dec 4;7(12):10189–208. doi: 10.3390/nu7125527 (PMC4690079; doi:10.3390/nu7125527)
Supplement: Supplementary file 1 [file nutrients-07-05527-s001.docx]

**Supplementary Materials: Optimal Vitamin D Supplementation Doses that Minimize the Risk for Both Low and High Serum 25-Hydroxyvitamin D Concentrations in the General Population**

**Paul J. Veugelers *, Truong-Minh Pham and John Paul Ekwaru**


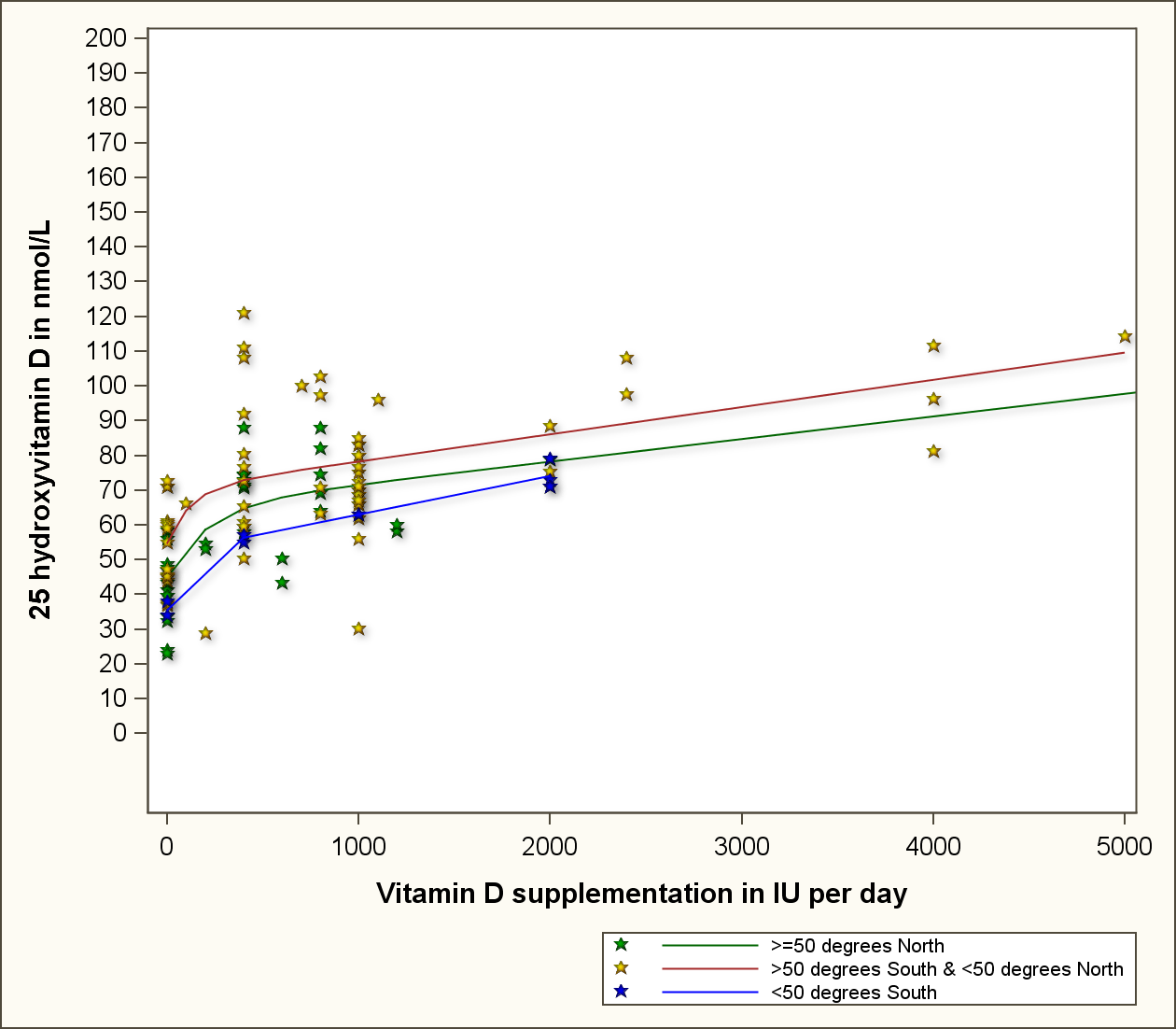


**Figure S1.** Mean serum 25(OH)D concentrations by vitamin D supplementation derived from published means and standard deviations stratified by latitude. Note: No studies were available that examined doses above 2000 IU per day in locations above 50° N and below 50° S.


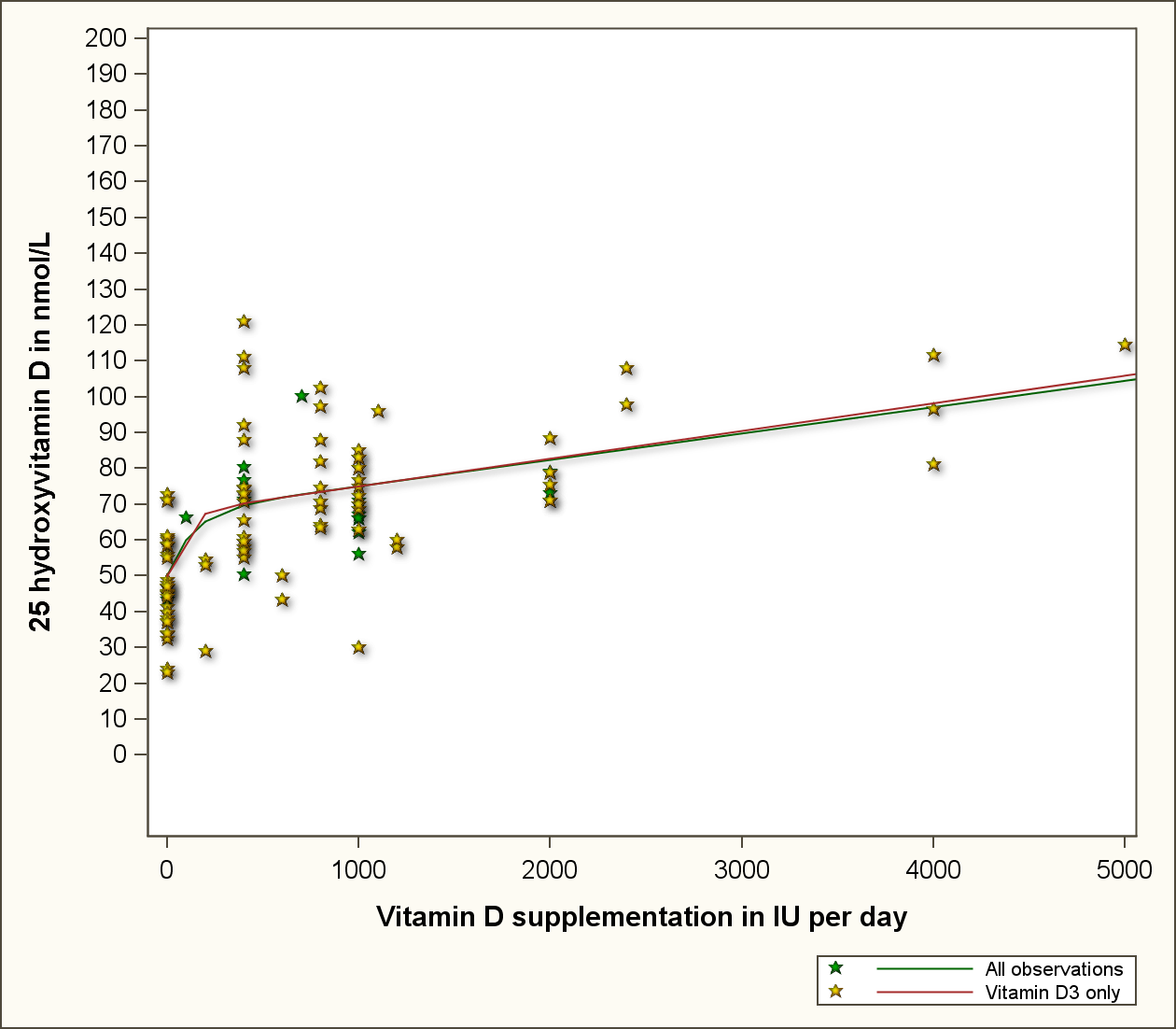


**Figure S2.** Mean serum 25(OH)D concentrations by vitamin D supplementation derived from published means and standard deviations stratified by vitamin D type. Note: Too few studies had examined vitamin D2 to allow for a direct comparison of vitamin D2 with vitamin D3.


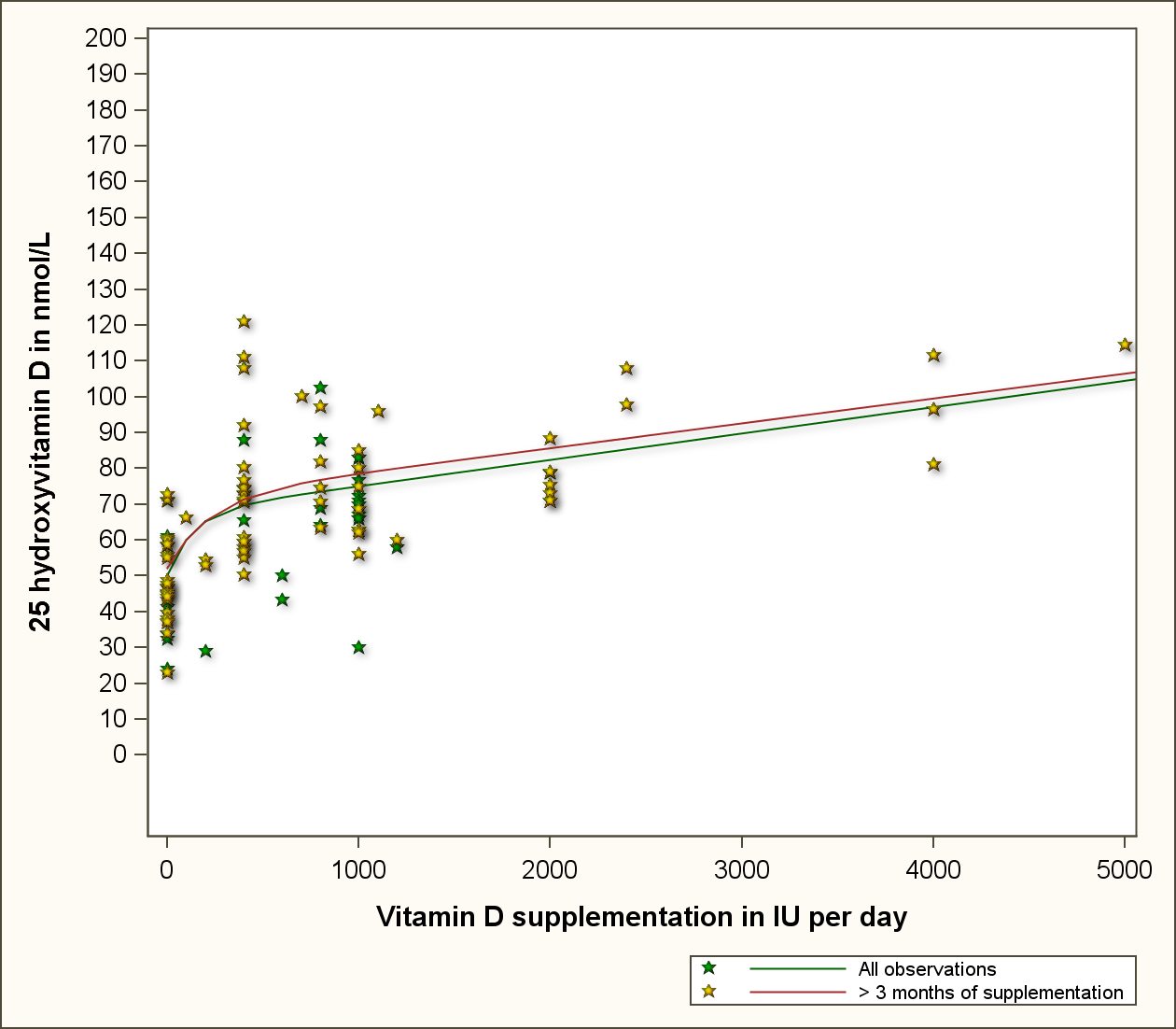


**Figure S3.** Mean serum 25(OH)D concentrations by vitamin D supplementation derived from published means and standard deviations stratified by period between commencing vitamin D supplementation and assessing serum 25(OH)D concentrations. Note: Too few studies with a short period between commencing supplementation and assessment of serum 25(OH)D concentrations were available to allow for stratification by the length of this period.
